# Call to introduce environmental preventive medicine courses to the medical curriculum. An initial experience of an education program at the Faculty of Medicine of Nice, University of Côte d'Azur

**DOI:** 10.3389/fmed.2024.1412674

**Published:** 2025-01-09

**Authors:** Patrick Fénichel, Emiko Todaka, Ruth A. Etzel, Chang-Chuan Chan, Robert Barouki, Nicolas Chevalier, Jean-Baptiste Fini, Kirsten R. Poore, Kou Sakabe, Valerie Siroux, Midori Yamamoto, Chisato Mori

**Affiliations:** ^1^Department of Endocrinology, Diabetology and Reproduction, University of Côte d'Azur, Nice, France; ^2^Center for Preventive Medical Sciences, Chiba University, Chiba, Japan; ^3^Milken Institute School of Public Health, George Washington University, Washington, DC, United States; ^4^College of Public Health, National Taiwan University, Taipei, Taiwan; ^5^Inserm UMRS-1124 Université de Paris, Paris, France; ^6^Laboratoire PHYMA, MNHN, UMR 7221, Paris, France; ^7^Faculty of Medicine, University of Southampton, Southampton, United Kingdom; ^8^Inserm U1209 CNRS UMR 5309 Team of Environmental Epidemiology Applied to the Development and Respiratory, Health Institute for Advanced Biosciences, Université Grenoble Alpes, La Tronche, France

**Keywords:** environmental medicine, education program, medical schools, endocrine disruptors, preventive medicine

## Introduction

Non-communicable diseases, including neuro-developmental, reproductive, metabolic, cardiovascular, oncologic, allergic/immune, and neurodegenerative diseases, have become more frequent worldwide than infectious diseases (1). Since 1990, there has been a marked shift in the global burden due to years lived with disability from communicable diseases to non-communicable diseases and injuries (2). Non-communicable diseases kill 41 million people each year, equivalent to 74% of all deaths globally (3). Adults with NCDs had an increased risk of death during the COVID-19 pandemic and could be similarly at risk during future epidemics (4). Diseases of pure genetic origin with single germinal mutations are found only in a very small proportion of chronic diseases. Currently, chronic, non-communicable diseases are considered to result from a combination of genetic susceptibility and a variety of environmental exposures. Among these environmental exposures, air and chemical pollution play a major role, but lifestyle factors, nutrition, psychological stress, and socioeconomic conditions are also involved. To improve environmental exposure assessment, Christopher Wild proposed the concept of the exposome in 2005: “At its most complete, the exposome encompasses life-course environmental exposures (including lifestyle factors), from the prenatal period onwards” (5). The rapid environmental changes in the Anthropocene have caused harm to both human and veterinary health, which can no longer be isolated from the harms to Earth's environment, including air (6), chemical (7), and ocean (8) pollution, as well as the decline in biodiversity and climate change—issues that are addressed through the “One Health” approach (9).

Despite calls from researchers (10), the curriculum of most medical schools in the world does not yet incorporate concepts concerning the impact of the environment on human health and the consequences for public health policy and preventive medical practice (11). Medical students themselves have recognized this, as recently reported in a French national survey, which showed they considered the topic of preventive and environmental medicine to be insufficiently taught (12). While there have been some student-led initiatives (13), we aimed to establish a faculty-led education program on environmental preventive medicine in the medical school curriculum. This medical field includes individual patient care, population health management, and non-medical risk management interventions, such as product substitution and patient education. The field draws on both One Health (9) and Planetary Health (14) concepts (15). We report here our positive experience in enhancing students' understanding of environmental preventive medicine.

## Background to the environmental preventive medicine program

An official collaboration between Chiba University in Japan and the University of Côte d'Azur in Nice, France, was established in 2016, following initial meetings between the teams of Professor Chisato Mori, Director of the Center for Preventive Medical Sciences at Chiba University, and Professor Patrick Fénichel, Director of the Endocrine and Reproductive Medicine Department of the University Hospital of Nice (University of Côte d'Azur). After initially collaborating around the theme of human exposure to endocrine-disrupting chemicals, this expanded to a wider goal of exchanging scientific research and educating students about preventive environmental medicine, both in Nice and beyond. An education course was designed after extensive discussions that would cover topics that newly trained doctors would likely face in their practices, all core to the practice of environmental preventive medicine (16). There was a particular focus on the effect of environmental exposures during critical periods of early life as a foundation to reduce the lifetime risk of non-communicable disease.

Two initial iterations of this course took place in 2017 and 2018 at the University Hospital of Nice, which included a small group of students from Chiba University and a larger cohort of approximately 80 Year 3–5 medical students from the University of Côte d'Azur, who attended on a voluntary basis. In 2018, a brief student evaluation of the course was undertaken that showed it was positively received (overall approval rating of 4.4 out of 5, *n* = 26 respondents).

Informed by these initial iterations of the education course, since 2022, all students at the University of Côte d'Azur's Faculty of Medicine have benefited from environmental health teaching. In their first year, students complete 6 h of teaching covering global concepts such as One Health, major health issues linked to environmental issues, methodologies for quantifying hazards and risks, and the concept of endocrine disruptors. In Year 3, students receive 12 h of teaching focused on environmental health and climate change. These efforts have been shaped by a requirement within the French national program for medical faculties to include environmental medicine in Year 2 of the curriculum.

## Environmental preventive medicine course in 2023

The most recent edition of the environmental preventive medicine course took place in November 2023 at the Faculty of Medicine of the University of Côte d'Azur in Nice. This course was designed for final year (Year 6) students, who thus had a strong background of scientific knowledge and were better equipped to embed environmental medicine into their clinical experience. In addition, following a proposal from the Faculty of Medicine's Dean, the course was made mandatory, with students registering their attendance twice a day before each half-day session. Those who were not able to attend were required to take a test after watching a recording of the course. Students completing the course were at an advantage in the final competition, which determines their specialty choice for the future. A cohort of 180 students, along with 10 invited students from Chiba University, attended the course, which was given in English with simultaneous translation to French throughout.

The program of the course is shown in Table 1 and includes 30-min lecture-style presentations followed by 15 min of questions and discussion with the students. A pre- and post-course knowledge quiz was delivered via an online platform (Vevox) based on the topics in each presentation (12 multiple-choice questions) at the start and end of the course. The learning outcomes of the course were to ensure students understood several environmental preventive medicine concepts, including a complete environmental history, environmental injustice, the Developmental Origins of Health and Disease (DOHaD) (17), the role of epigenetics in the occurrence of chronic diseases, epidemiological transition, exposome (18), and mechanisms of endocrine disrupting chemicals (19). All the topics were supported by epidemiological and experimental studies and were delivered by a multidisciplinary team of clinicians and scientists. Our objective was to help students understand the physio-pathological origins of non-communicable diseases, the concepts of One Health and planetary health, and the importance of education and prevention at critical periods of development (20, 21). This involved a discussion of the individual and collective public health responsibilities, as well as the role of general practitioners as public health actors at the preventive medical level (22). The expert international speakers provided students with a practical understanding of the environmental health problems experienced by different cultures and societies and the common threats to human health caused by environmental changes. This allowed the students to think more broadly than through their cultural lens. We believe this approach is necessary to give students the cultural sensitivity to understand environmental injustice across generations, race, wealth, and poverty, in the Global North and Global South.

**Table 1:** Program of the “Third International Education Course on Environmental Preventive Medicine” co-organized by Chiba University and University of Côte d'Azur (Faculty of Medicine of Nice) 22–23 November 2023.

**Session title**	**Invited speaker**
***Day 1***	
Greetings from Prof. Jean Dellamonica, the Dean of the Faculty of Medicine, University of Côte d'Azur	
Greetings from Dr. Richard Chemla, Deputy City Mayor of Nice	
Opening remarks by Prof. Chisato Mori, Center for Preventive Medical Sciences, Chiba University	
Online quiz of student knowledge before the course	Dr. Kirsten Poore
**Session 1: “Early life environmental determinants of health in later life”**	
“Developmental Origins of Health and Disease (DOHaD) and Epigenetics”	Dr. Kirsten Poore
“Children are not little adults”	Prof. Ruth Etzel
**Session 2: “Pollutants and human health”**	
“Learning from historical disaster and tragic events: Minamata Disease, Seveso, etc.”	Prof. Emiko Todaka
“Endocrine Disrupting Chemicals (EDCs)”	Prof. Robert Barouki
“Agricultural pesticides and human health”	Prof. You Sakabe
**Session 3: “Modern issues of human health”**	
“Neurodevelopmental diseases”	Prof. Jean-Baptiste Fini
“Recent increase of metabolic syndrome including obesity and diabetes”	Prof. Nicolas Chevalier
“Air Pollution and Health”	Prof. Chang-Chuan Chan
***Day 2***	
**Session 4: “Emerging health issues and environmental exposures I”**	
“Environmental Epidemiology, Exposome and Biomarkers”	Prof. Valerie Siroux
“One Health”	Prof. Ruth Etzel
“Plastic pollution and human health”	Prof. Patrick Fénichel
**Session 5: “Emerging health issues and environmental exposure II”**	
“Climate change and health”	Prof. Ruth Etzel
“Chemical sensitivity”	Prof. Kou Sakabe
“Japan Environment and Children's Study by the Ministry of the Environment of Japan”	Dr. Midori Yamamoto
**Session 6: “Prevention and intervention” discussion by all the participants**	
“How to prevent parental–fetal exposure to endocrine disruptors? The pre-conception consultation”	Prof. Patrick Fénichel
**Final discussion by all the lecturers and students**	
Online quiz of student knowledge after the course	Dr. Kirsten Poore
Student online evaluation of the course	Dr. Kirsten Poore
Closing remarks	Prof Chisato Mori

## Feedback about the environmental preventive medicine course in 2023

The majority of students stayed until the end of the course; they remained attentive and interactive, asking many pertinent questions at the end of each lecture. They explained that they had not received extensive teaching on these topics before. However, we were impressed by their ability to understand, integrate, and synthesize these new data and concepts and to realize the implications from both individual and collective points of view. When comparing student knowledge before and after the course, the percentage of students answering each question correctly was increased for all questions, showing the effectiveness of the teaching and learning during the course (Table 2).

**Table 2:** Effect of the environmental preventive medicine course on student knowledge of topics presented.

**Question topic**	**Pre-course results (% students correct)**	**Post-course results (% students correct)**
Postnatal lung development	24%	70%
Vulnerability of children to air pollution	86%	90%
Cause of Minamata disease	33%	92%
Health outcome associated with childhood exposure to lead	28%	46%
Chemical compound linked to type 2 diabetes	0.7%	13%
Childhood obesity associated with bisphenol A exposure	23%	43%
Health outcome associated with PM2.5 exposure	34%	93%
Worldwide number of deaths from COVID-19	40%	41%
Organizations leading the One Health approach	29%	46%
Alternative sources of power generation	40%	80%
Effect of sea level rise	39%	84%
Importance of folic acid supplementation pre-conception	36%	71%

Multiple choice (one best answer) questions were presented to students using an online polling website (Vevox) immediately before (pre-course, without revealing correct answers) and immediately after (post-course, revealing and briefly discussing correct answers) the course. N = 149 participants.

All students were asked for their evaluation of the course on completion; 38 students provided numerical (on a scale of 1 to 5) and qualitative feedback. Students found the course intellectually stimulating (score 4.4 out of 5) and clinically relevant (score 4.4 out of 5) and would recommend the course to other students (score 4.7 out of 5). Students clearly understood the aims of the course (score 4.8 out of 5) and found the material covered was appropriate (score 4.6 out of 5), given their prior knowledge as Year 6 students. The overall course rating was 4.6 out of 5 (95% giving a rating of 4 or 5). Crucially, 74% of students who responded agreed that the course would influence their future practice as a doctor.

We believe that bringing French students together with Japanese students for this course helped to create a collaborative learning environment to foster the development of students who will be equipped to respond to global problems through effective collaboration and transdisciplinarity (23). Although some students found the course too intensive at times, with some sessions too long and overly scientific in nature, their level of interaction with the course suggested they were conscious it was important to them as future doctors. We explained to them that we deliberately presented original epidemiological and experimental data during the lectures to support the conclusions, following an evidence-based medicine approach. Students suggested that the course should dedicate more time to providing practical advice and methods to educate their patients. Some students were worried about not being able to advise patients in their medical practice due to a lack of time, practical solutions, and knowledge. We understood that they wanted more practical advice, patient-focused sources of information, questionnaires, applications, and pedagogical methods to use with their patient consultations.

Students were also conscious that the course content was important for their own lives and humanity in general, developing philosophical reflections and questions. Some even questioned whether it was reasonable or pertinent to start a family given the many environmental and climate pressures facing them. They could not understand why politicians and policymakers did not care more about the link between the environment and health.

## Our ongoing education approach

The feedback from the 2023 course and its earlier iterations were overwhelmingly positive, and this is now shaping the future of the medical curriculum at the University of Côte d'Azur's Faculty of Medicine. In addition to content in Years 1–3, in 2024, a social responsibility and the eco-design of healthcare theme has been added to the curriculum (15 h of workshop teaching). In Year 4, students are trained to provide practical advice on the prevention and promotion of environmental health for use with secondary school students and staff in vocational training, directly responding to students' requests for practical advice to help their patients. In addition, the faculty is proposing 6–12 h of teaching on environmental medicine in Year 6, based on the model of our 2023 course, to reinforce specific knowledge they have achieved throughout the curriculum.

All medical schools have a crowded curriculum, and there is considerable competition for time to be allocated to new topics. We argue that future doctors need to be prepared to deal with the health challenges of our changing environment. Including new content about environmental preventive medicine could be done using various methods, such as the course presented here, but need not be burdensome for students. Others have used an “infusion” approach to gently reinforce concepts over many years of study and in a variety of contexts (24). This is aligned with the new approach at the University of Côte d'Azur, which begins in Year 1 and is reinforced in Year 6 following recent national recommendations.

## Conclusion

We conclude that this original and innovative teaching experience should not only be a recommendation but an absolute necessity to introduce and develop environmental preventive medicine into the curriculum in worldwide medical faculties. We have proposed one model based on a final-year course that has been effective and well-received. This may have three positive general consequences for future healthcare practitioners: (1) to bring more attention to the impact of the environment on health in clinical practice, (2) to give pertinent preventive medical advice to patients, and (3) to enable future specialists in environmental health to inform policymakers on ways to reduce harmful environmental exposures at the local, national, and international levels. We believe this is a universal competence that all medical students need before they enter clinical practice in our increasingly complex and interconnected world.
