# Supplementary material for: Call to introduce environmental preventive medicine courses to the medical curriculum. An initial experience of an education program at the Faculty of Medicine of Nice, University of Côte d'Azur
Source: Front Med (Lausanne). 2025 Jan 9;11:1412674. doi: 10.3389/fmed.2024.1412674 (PMC11755413; doi:10.3389/fmed.2024.1412674)
Supplement: Supplementary file 1 [file Table_1.DOCX]

**Table 1:** Program of the “Third International Education Course on Environmental Preventive Medicine” co-organized by Chiba University and University of Côte d’Azur (Faculty of Medicine of Nice) 22^nd^– 24th November 2023

**Day 1**

Greeting from Professor Jean Dellamonica, the Dean of Faculty of Medicine, University of Côte d’Azur

Greeting from Dr. Richard Chemla, Deputy City Mayor of Nice

Opening remarks by Professor Chisato Mori, Center for Preventive Medical Sciences, Chiba University

Pre-evaluation of the knowledge by Smartphone by Prof. Kirsten Poore

**Session 1:** “**Early life environmental determinants of health in later life**”

“Developmental Origins of Health and Disease (DOHaD) and Epigenetics” by Prof. Kirsten Poore)

“Children are not little adults” by Prof. Ruth Etzel

**Session 2: “Pollutants and human health”**

“Learning from historical disaster and tragic events: Minamata Disease, Seveso, etc.” by Prof. Emiko

“Endocrine Disrupting Chemicals (EDCs)” by Prof. Robert Barouki

“Agricultural pesticides and human health” by Prof. Kou Sakabe

**Session 3: “Modern issues of human health”**

“Neurodevelopmental diseases” by Prof. Jean-Baptiste Fini

“Recent increase of metabolic syndrome including obesity and diabetes” by Prof. Nicolas Chevalier

“Air pollution and Health” by Prof. Chang-Chuan Chan

**Day 2**

**Session 4: “Emerging health issues and environmental exposures I”**

“Environmental Epidemiology, Exposome and Biomarkers” by Prof. Valerie Siroux , (online)

“One Health” by Prof. Ruth Etzel

“Plastic pollution and human health” by Professor Patrick Fénichel

**Session 5: “Emerging health issues and environmental exposure II”**

“Climate change and health” by Prof. Ruth Etzel

“Chemical sensitivity” by Prof. Kou Sakabe

“Japan Environment and Children’s Study by the Ministry of the Environment of Japan” by Dr. Midori Yamamoto

**Session 6: “Prevention and intervention” discussion by all the participants**

“How to prevent parental-fetal exposure to endocrine disruptors?

The pre-conception consultation” by Prof. Patrick Fénichel

**Final Discussion by all the lecturers and the students**

Evaluation of the course by Smartphone by Prof. Kirsten Poore

Closing remarks by Prof. Chisato Mori
